# The Clinical Aspects and Prognostic Factors Concerning Survival in Patients With Recurrent Cervical Cancer After Radical Hysterectomy and Adjuvant Chemoradiotherapy

**DOI:** 10.3389/fonc.2021.782403

**Published:** 2022-01-21

**Authors:** Hui-Ting Zhu, Wen-Juan Yan, Yu-Hua Gao

**Affiliations:** ^1^ Department of Gynecology, Liaoning Cancer Hospital & Institute, Cancer Hospital Of China Medical University, Shenyang, China; ^2^ Department of General Surgery, General Hospital of Eastern Theater Command, People’s Liberation Army (PLA), Nanjing, China

**Keywords:** recurrent cervical cancer, prognostic factors, survival, radical surgery, radiotherapy, chemotherapy

## Abstract

**Purpose:**

To investigate the recurrence patterns and prognostic factors of patients with recurrent cervical cancer after radical hysterectomy with node dissection (RHND) followed by adjuvant radiotherapy (RT)/concurrent chemoradiotherapy (CCRT).

**Methods:**

The medical records of 153 patients with pre-operative International Federation of Gynecology and Obstetrics stage IB-IIA cervical cancer, who were treated with RHND followed by adjuvant RT/CCRT at the Liaoning Cancer Hospital between January 1, 2012 and May 31, 2018, were retrospectively analyzed.

**Results:**

The median disease progression-free survival time was 16 months, and 75.2% (115/153) of patients had a relapse within two years. The survival of patients with multi-site relapse was significantly lower in comparison to those with relapse in a single site (p < 0.001). The survival rate of patients with distant metastasis (DM) and combined recurrence (DM with localregional recurrence [LR]) was significantly lower than that of patients with only LR (p = 0.006, p < 0.001). Furthermore, the survival rate of patients with combined recurrence was significantly lower than that of patients with only DM (p = 0.046). Multivariate analysis showed that resection margin involvement, para-aortic and common iliac lymph node metastasis, DM, no treatment after disease relapse, and early disease relapse were independent prognostic factors associated with poor survival.

**Conclusion:**

Most of the cervical cancer patients who received initial RHND followed by adjuvant RT/CCRT had a relapse within two years. Resection margin involvement, para-aortic and common iliac lymph node metastasis, DM, no treatment after recurrence, and early disease relapse were found to be prognostic factors in patients with recurrent cervical cancer after RHND followed by adjuvant RT/CCRT.

## Introduction

Cytological screening has substantially reduced the incidence rate and mortality rate of cervical cancer, but cervical cancer is still the fourth most common malignancy in women (1, 2). When cervical cancer is detected at an early stage (stages IB-IIA), based on the 2009 International Federation of Gynecology and Obstetrics (FIGO) staging system, radical hysterectomy with node dissection (RHND) is the preferred surgical treatment. Postoperative adjuvant radiotherapy (RT) or concurrent chemoradiotherapy (CCRT) is also recommended, depending on the risk factors as evaluated in postoperative histopathological examinations (3, 4).

Intermediate-risk factors include large tumor size, lymphovascular space invasion (LVSI), and deep cervical interstitial infiltration (5, 6). High-risk factors include lymph node metastasis, parametrial invasion, and resection margin involvement (7, 8). The existence of risk factors is associated with a higher recurrence rate and poor survival outcome in patients with early cervical cancer. These patients can benefit from postoperative RT or CCRT, which can prolong the disease progression-free survival (PFS) time and overall survival (OS) time (7).

Considering the relatively short survival time of patients with recurrent cervical cancer, it is vital to identify the prognostic factors for recurrent cervical cancer after the initial treatment. However, the clinical features and the effect of each risk factor on the recurrent cervical cancer patient is not well-known. In addition, according to the latest International Federation of Gynecology and Obstetrics (FIGO) staging system, lymph node metastasis is defined as stage IIIC, and the prognosis of these patients after radical surgery and adjuvant RT/CCRT is not very clear.

Therefore, the purpose of this retrospective study was to identify the recurrence pattern and prognostic factors of patients with recurrent cervical cancer after initial treatment with RHND followed by adjuvant RT/CCRT.

## Patients And Methods

### Study Population

The medical records of recurrent cervical cancer patients who were initially treated with RHND and adjuvant RT/CCRT and registered between January 1, 2012 and May 31, 2018 in Liaoning Cancer Hospital were retrospectively analyzed.

The inclusion criteria were patients with histologically diagnosed cervical cancer, preoperative FIGO stage IB-IIA disease, and no history of neoadjuvant chemoradiotherapy (CRT), who had received RHND and postoperative pelvic RT (dose ≥40 Gy) with or without CCRT.

Pre-treatment examinations included gynecological examination, blood routine examination, blood biochemical examination, urine routine examination, squamous cell carcinoma antigen (SccAg), chest and abdomen computed tomography (CT), pelvic magnetic resonance imaging (MRI), or positron emission tomography/computed tomography (PET/CT). Cystoscopy and colonoscopy were performed when the bladder and rectum were suspected of being involved. The pathological reports that were analyzed retrospectively included histological subtype, pathological differentiation degree, tumor size, LVSI, interstitial infiltration depth, number of lymph nodes dissected, number of positive lymph nodes in each site, parametrial invasion, and margin resection involvement.

### Treatment

All the patients initially received adjuvant RT/CCRT after RHND. In accordance with the National Comprehensive Cancer Network (NCCN) consensus guidelines, patients with any high-risk factor received adjuvant pelvic RT + platinum-based concurrent chemotherapy +/− vaginal brachytherapy. Patients with only moderate risk factors received adjuvant pelvic RT +/− platinum-based concurrent chemotherapy +/− vaginal brachytherapy, according to the Sedlis standard. Conformal RT or intensity-modulated RT started within 4–6 weeks of radical surgery. According to the clinical target volume (CTV) guidelines of RT tumor groups for the whole pelvis, the CTV included the parauterine area, the upper vagina, and the pelvic lymph drainage area (the common iliac blood vessel, internal and external iliac blood vessel, obturator lymph node, and presacral lymph node areas). In addition, the para-aortic lymph node area was included when para-aortic lymph node metastasis had occurred.

A dose of 44.0–50.4 Gy in 22–28 fractions (1.8– 2.0 Gy/day) was delivered to at least 95% of the planning clinical target volume (PCTV). Patients received radiotherapy in 5 fractions per week over 4.5–6 weeks. Intracavitary brachytherapy was used for patients with vaginal lesions close to the resection margin (≤5 mm) or with a positive resection margin. The total dose was 10–18 Gy in 2–6 fractions. Dose limits for organs at risk were as follows: spinal cord D 0.1cc ≤ 45 Gy, small intestine D 2cc ≤ 54 Gy, bladder D 50% ≤ 45 Gy, rectum D 50% ≤ 45 Gy.

The concurrent chemotherapy, based on cisplatin, included weekly cisplatin (30–40 mg/m^2^) for 4–6 courses or 3–4 courses of paclitaxel and cisplatin/carboplatin every three weeks.

The treatment of recurrent cervical cancer depended on the initial treatment methods, the site of recurrence, and the patient’s physical condition. The treatment modes included operation, radiotherapy, chemotherapy, comprehensive treatment, immunotherapy, and palliative care. Surgery referred to simple lesion resection, which was performed in patients with isolated pelvic or vaginal recurrence. Surgery was defined as therapeutic surgery and did not include symptomatic surgery. Palliative care was defined as symptomatic supportive care.

### Follow-Up Evaluations

Patients received the first follow-up evaluation one month after the end of treatment, then, every three months in the first two years, every six months in the third to fifth years, and once a year after five years. The main follow-up examinations were a gynecological examination, an SccAg, chest and abdomen CT, and pelvic MRI. PET/CT was recommended only when disease relapse was suspected.

Locoregional relapse (LR) was defined as any disease relapse in the radiation field, including the vaginal stump and pelvic lymph node area below the aortic bifurcation. Distant metastasis (DM) was defined as disease relapse outside the radiation field. PFS was defined as the time from the day of surgery to disease relapse or the latest follow-up, and OS after recurrence was defined as the time from when disease relapse was diagnosed to cervical cancer-specific death or the latest follow-up. In this study, death after recurrence was defined as specific death of cervical cancer, excluding death due to any other reason.

### Statistical Analysis

The risk factors for specific death of recurrent cervical cancer were analyzed. The categorical variables were age (a continuous variable), type of surgical approach, disease early relapse (recurrence occurred within six months of the day of surgery), number of recurrent sites, recurrence site, histological diagnosis, tumor size, pathological differentiation degree, LVSI, interstitial infiltration depth, parametrial invasion, resection margin involvement, pelvic lymph node metastasis, and para-aortic lymph node metastasis.

A chi-square test or Fisher exact test were used for the univariate analysis of the categorical variables. The variables found to have statistical significance in the univariate analysis were used in the subsequent multivariate analysis. Multivariate analysis was performed with the Cox proportional hazard model. The Kaplan–Meier method and log-rank test were used to evaluate the influence of risk factors on the survival rate after disease relapse. Results showed a 95% confidence interval for the risk ratio (RR). A value of p < 0.05 was considered to be statistically significant.

## Results

### Patient Characteristics

Between January 2012 and May 2018, a total of 415 patients with biopsy-proven cervical cancer were treated with RHND followed by RT/CCRT, and 153 of them suffered a relapse and were enrolled in this study.

The clinicopathological characteristics of the recurrent cervical cancer patients are presented in **Table 1**. The median age was 47 years old (range: 29–67 years), and the main histological type was squamous cell carcinoma (92.8%, 142/153). Deep interstitial infiltration was the most common risk factor (93.5%, 143/153), followed by LVSI (77.1%, 118/153) and lymph node metastasis (43.8%, 67/153). Twenty-one patients had a positive resection margin (13.7%, 21/153). Among the 153 patients, 82 had high-risk factors, 76 having a single high-risk factor and the remaining 6 two high-risk factors, while 131 patients received RT/CCRT after surgery, and 22 of them only received RT.

**Table 1:** Basic characteristics of recurrent cervical cancer patients after initial treatment.

	Number	Proportion (%)
Age (years old)		
≤50	96	62.7
>50	57	37.3
Postoperative FIGO staging (2018)		
IA	0	0
IB	59	38.6
IIA	26	17.0
IIIC1	45	29.4
IIIC2	23	15.0
Histological subtype		
Squamous cell carcinoma	142	92.8
Adenocarcinoma	9	5.9
Adenosquamous carcinoma	2	1.3
Lesion diameter		
<4cm	94	61.4
≥4cm	59	38.6
Pathological differentiation degree		
High/High-medium	34	22.2
Medium	52	34.0
Low/Medium-Low	67	43.8
Interstitial infiltration depth		
<1/2	10	6.5
≥1/2	143	93.5
Lymphovascular space invasion		
Yes	118	77.1
No	35	22.9
Parametrial infiltration		
Yes	0	0
No	153	100
Resection margin involvement		
Yes	21	13.7
No	132	86.3
Lymph node metastasis		
Yes	67	43.8
No	86	56.2

### Recurrence Pattern

**Table 2** shows the patterns of relapse and treatment mode of recurrent cervical cancer patients. In total, 153 patients had disease relapse in this study, and 105 of them (105/153, 68.6%) had DM. Of these patients, 67 (67/153, 43.8%) only had DM, while 38 (38/153, 24.8%) had DM with LR. The other 48 patients (48/153, 31.4%) only had LR.

**Table 2:** Recurrence patterns and treatment mode of recurrent cervical cancer patients.

	Patients No.	Proportion (%)
Disease PFS (month)		
≤6	38	24.8
>6	115	75.2
Recurrence site		
LR (pelvic sidewall)	10	6.5
LR (central pelvis)	38	24.8
DM	67	43.8
DM+LR	38	24.8
Number of recurrent sites		
Single	84	54.9
Multiple	69	45.1
Treatment after recurrence		
Chemotherapy	73	47.7
Radiotherapy	3	2.0
Surgery	7	4.6
Chemoradiotherapy	35	22.9
Surgery+chemotherapy/chemoradiotherapy	19	12.4
Chemotherapy+targeted therapy	5	3.3
Immunotherapy	3	2.0
Palliative therapy	8	5.1

The lungs were the most common DM site (55/105, 52.4%), followed by the lymph nodes (44/105, 41.9%) and bone (34/105, 32.4%). Liver metastasis accounted for 19.0% (20/105), and other sites accounted for 8.6% (9/105). Of the patients with DM, 43.8% (46/105) had multi-site relapse.

This study found that the median disease PFS time was 16 months (range: 5–70 months), 75.2% (115/153) of patients had recurrence within two years, and 24.8% (38/153) had early disease relapse (recurrence occurred within 6 months of surgery).

The treatment after disease relapse included palliative chemotherapy in 73 cases (47.7%), palliative RT in 3 cases (2%), surgery in 7 cases (4.6%), chemotherapy combined with RT in 35 cases (22.9%), surgery combined with chemotherapy/RT in 19 cases (12.4%), chemotherapy combined with targeted therapy in 5 cases (3.3%), immunotherapy in 3 cases (2%), and palliative care in 8 cases (5.1%).

### Survival Analysis

This study further analyzed the survival outcome of cervical cancer patients after recurrence although five patients were eventually lost during follow-up. The median follow-up time was 19 months (5–55 months). The median survival time after disease relapse was 26 months (range: 10–54 months) and 18 months (range: 5-55 months) in patients with only LR relapse and only DM, respectively, whereas the median survival time of patients with combined recurrence was 14 months (range: 5–33 months).

**Figure 1:**
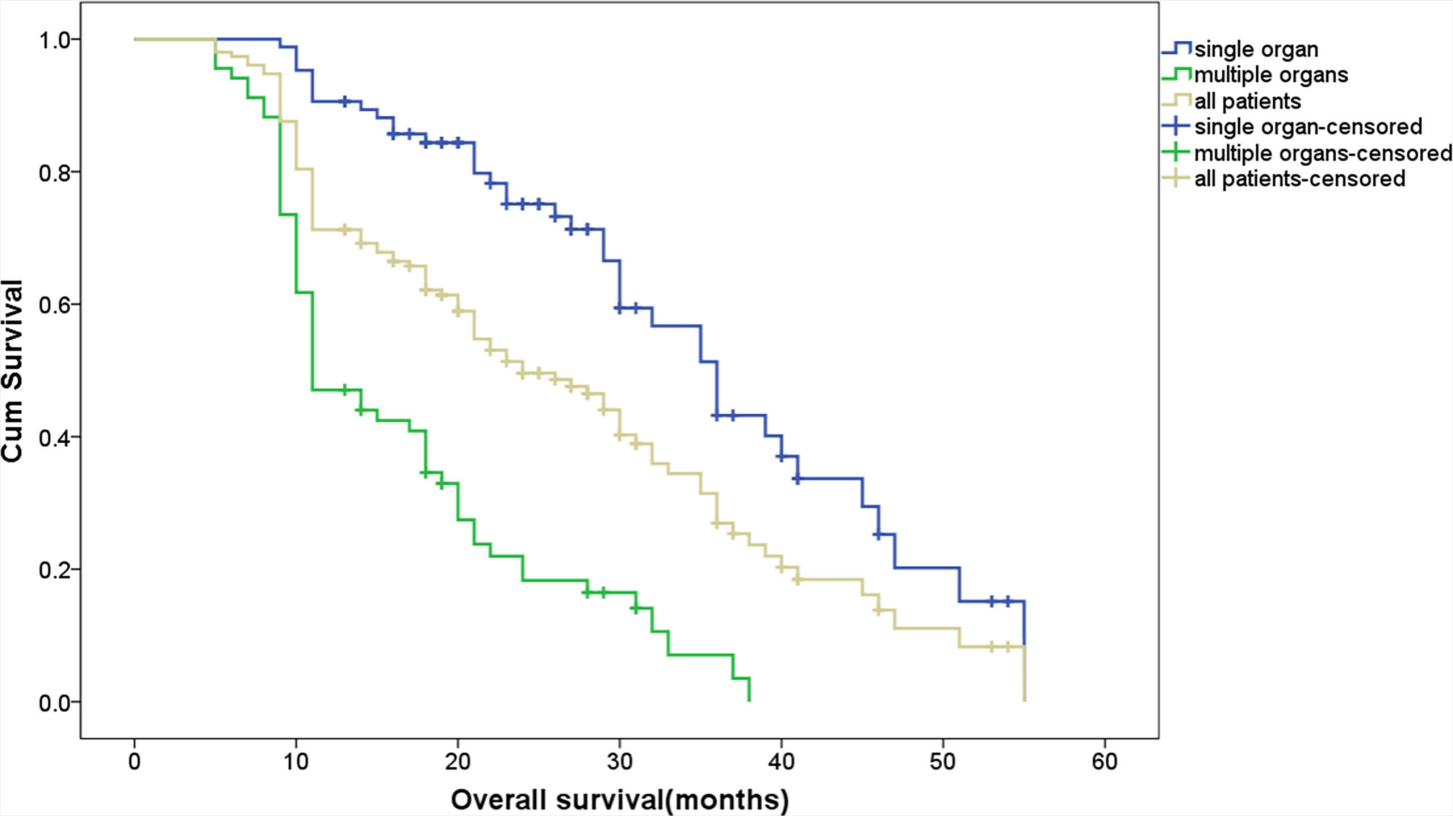
Overall survival comparing single site and mult-site relapse.

**Figure 2:**
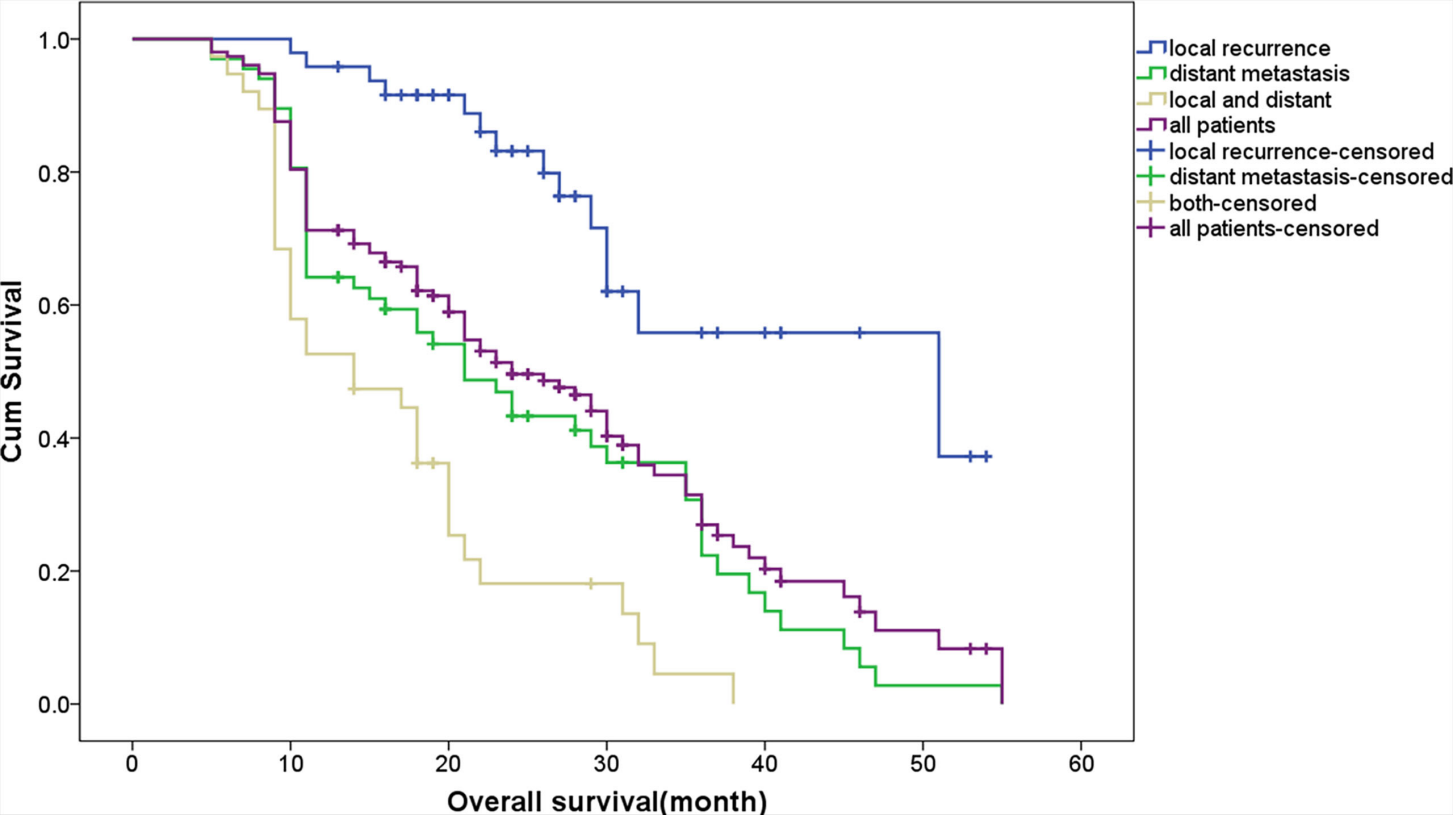
Overall survival comparing different recurrence patterns.

The survival curves according to the number of sites involved and the relapse patterns are shown in **Figures 1** and **2**, with 69 (45.1%, 69/153) patients being diagnosed as having multi-site involvement. The 1 -, 2 -, and 3-year survival rates of these patients were 47.06%, 14.71%, and 2.9%, respectively. The study indicated that the survival of patients with recurrences in multiple sites was significantly lower in comparison to those with recurrences in a single site (p < 0.001). In addition, statistical analysis showed that there was a significant difference in the survival rate between different recurrence patterns (p < 0.001). The survival rate of patients with only DM and with combined recurrence was significantly lower than that of patients with only LR (p = 0.006, p < 0.001). Furthermore, the survival rate of patients with combined recurrence was significantly lower than that of patients with only DM (p = 0.046). A stratified analysis was then made according to the different recurrence patterns, and the prognostic factors of the three recurrence patterns were analyzed (**Table 3**). Multivariate analysis showed that the influencing factor of survival in patients with LR alone was disease stage, the influencing factors of DM alone were para-aortic lymph node metastasis and pathological differentiation, and the influencing factors of combined recurrence were common iliac lymph node metastasis and resection margin involvement.

**Table 3:** The prognostic factors of LR, DM, DM+LR.

Variable	P value	95%CI	HR
Only LR			
Postoperative FIGO staging	0.028		
IB			Reference
IIA	0.757	0.356-4.013	0.863
IIIC1	0.107	0.773-14.001	3.289
IIIC2	0.003	2.155-40.220	9.310
Only DM			
Para-aortic lymph node metastasis	0.023		
No			Reference
Yes		1.125-5.030	2.379
Pathological differentiation degree	0.042		
Low/Medium-Low			Reference
Medium	0.016	0.205-0.848	0.416
High/High-medium	0.125	0.133-1.178	0.413
DM+LR			
Common iliac lymph node metastasis	0.000		
No			Reference
Yes		2.377-15.944	6.156
Resection margin involvement	0.005		
No			Reference
Yes		1.440-7.606	3.309

**Tables 4** and **5** present the details of the univariate and multivariate analysis. The univariate analysis showed that disease stage, operation mode, tumor size, pathological differentiation degree, positive resection margin, positive lymph nodes in pelvic cavity, positive para-aortic lymph nodes, different metastatic sites, number of involved sites, treatment modes after disease relapse and early disease recurrence were associated with death after disease relapse. In multivariate analysis, the variables with statistical differences in the above univariate analysis were included in the Cox proportional hazard regression model. The results showed that the risk factors of death after disease relapse were positive resection margin, positive common iliac lymph node, positive para-aortic lymph node, different metastatic sites, no treatment after recurrence, and early disease recurrence. In addition, multivariate logistic regression analysis showed that the degree of pathological differentiation and common iliac lymph node metastasis were important influencing factors of early disease recurrence.

**Table 4:** Univariate analysis for factors associated with survival outcomes in patients with recurrent cervical cancer.

Variable	Median Survival Time (month)	*P* value
Age (years old)		0.459
≤50	19	
>50	20	
Postoperative FIGO staging (2018)		0.000
IB	22	
IIA	25.5	
IIIC1	18	
IIIC2	11	
Tumor size (cm)		0.045
<4	20.5	
≥4	17	
Histology		0.570
Squamous cell carcinoma	19.5	
Non-squamous cell carcinoma	18	
Pathological differentiation degree		0.000
Low/Medium-Low	14	
Medium	23	
High/High-medium	23.5	
Lymphovascular space invasion		0.300
Yes	19	
No	20	
Interstitial infiltration depth ()		0.314
<1/2	32	
≥1/2	19	
Resection margin involvement		0.012
Yes	20	
No	19	
Lymph node metastasis		0.000
Yes	14.5	
No	22	
Obturator lymph node metastasis		0.000
Yes	14.5	
No	22	
Internal and external iliac lymph node metastasis		0.000
Yes	11	
No	22	
Common iliac lymph node metastasis		0.000
Yes	11	
No	22	
Para-aortic lymph node metastasis		0.000
Yes	11	
No	20.5	
Recurrence site		0.000
Only LR	26	
Only DM	18	
DM+LR	14	
Number of recurrent s		0.000
Single	25	
Multiple	11	
Recurrence time (month)		0.000
≤6	11	
>6	22	
Treatment mode after disease relapse		0.000
Chemotherapy	18	
Chemoradiotherapy	25	
Surgery+chemotherapy	19.5	
Refuse treatment	10.5	

**Table 5:** Cox proportional hazard analysis for prognositc factors associated with survival outcomes in recurrent cervical cancer.

Variable	Overall survival		
	HR	95%CI	*P* value
Resection margin involvement			0.002
No	Reference		
Yes	2.728	1.455-5.115	
Common iliac lymph node metastasis			0.000
No	Reference		
Yes	4.516	2.702-7.546	
Para-aortic lymph node metastasis			0.023
No	Reference		
Yes	1.900	1.092-3.307	
Recurrence site			0.002
Only LR	Reference		
Only DM	2.054	1.054-4.006	0.035
DM+LR	3.895	1.846-8.217	0.000
Recurrence time ≤ 6 months			0.012
No	Reference		
Yes	2.148	1.185-3.894	
Treatment mode after disease relapse			0.032
Simply chemotherapy	Reference		
Chemoradiotherapy	0.716	0.418-1.228	0.225
Surgery+chemotherapy	0.487	0.212-1.119	0.090
Refuse treatment	2.635	1.134-6.121	0.024

## Discussion

Palliative chemotherapy is the main treatment for patients with recurrent cervical cancer. Individualized treatment also includes surgery, RT, targeted therapy, and immunotherapy. Although great efforts have been made to prolong the survival time of patients with recurrent cervical cancer in the past decades, the prognosis of these patients is still not optimistic. Some studies reported that the 1-year survival rate of recurrent cervical cancer was only 15–20% (9).

Minimally invasive surgery, open surgery and robotic surgery are mainly three surgery mode for primary radical hysterectomy. One study (10) enrolled 319 early stage cervical cancer patients, and randomly assigned patients to undergo minimally invasive surgery or open surgery, rates of postoperative adjuvant therapy (chemotherapy or radiotherapy) were similar in the two groups. The study indicated that minimally invasive radical hysterectomy was associated with lower rates of disease-free survival and overall survival than open abdominal radical hysterectomy among women with early-stage cervical cancer. However, it didn’t analyze the prognosist after disease relapse. Our study showed that in multivariate analysis, different surgery mode was not important influencing factor of prognosis after disease recurrence, although it showed significant difference in univariate analysis. Another study showed the equivalent survival figures of robotic and laparoscopic approaches to radical surgery of early stage cervical cancer patients (11). Our study was a retrospective study and the number of patients was not enough, maybe a large prospective randomized trial is needed to analyze the association between surgery mode and prognosis after disease relapse in cervical cancer.

This study analyzed the recurrence patterns and prognostic factors of patients with recurrent cervical cancer after radical surgery followed by adjuvant RT/CCRT. Most patients (75.2%) had disease relapse within two years. The median disease PFS was 16 months, which is consistent with the data reported in earlier studies (12–15), and 24.8% of patients were early disease recurrence cases. In addition, early disease recurrence was seen to be an independent risk factor for the prognosis of patients with recurrent cervical cancer (p = 0.012). NCCN guidelines suggest that follow-up evaluation should be conducted every 3 months within two years of initial treatment being completed, since early detection and early treatment may improve the prognosis of patients with early disease recurrence. Therefore, cervical cancer patients with high risk factors, proved by postoperative pathology, could be reexamined within half a year of the end of their initial treatment.

The study also indicated that the surgical variables of the independent risk factors leading to the poor survival of recurrent cervical cancer included a positive resection margin, positive common iliac lymph node, and positive para-aortic lymph node, which were all found to be high-risk factors in pathological tests. Cisplatin-based CRT is the standard treatment of postoperative adjuvant therapy for middle and high-risk cervical cancer patients. Additional treatment could be considered to prolong the survival time of patients with pathological high-risk factors, such as targeted therapy and systemic chemotherapy before/after RT. In another study (16), 34 cervical cancer patients with stage IIB-IIIB were treated with consolidation chemotherapy (paclitaxel combined with nedaplatin) for four cycles. The results showed that the complete remission rate was 88%, and the 2-year disease PFS rate was 82%. This study suggests that the concept of consolidation therapy may be feasible for cervical cancer patients with postoperative adjuvant CCRT if there are high-risk factors in surgical specimens.

In recent years, many studies have investigated the effect of positive pelvic lymph nodes on the survival outcome of cervical cancer. Shyu et al. (17) showed that the five-year survival rate of recurrent cervical cancer patients who underwent radical surgery with lymph node metastasis was significantly lower than that of patients with no lymph node metastasis. Meir et al. (18) suggested that lymph node metastasis is an independent risk factor for overall survival and disease progression-free survival of recurrent cervical cancer. The latest FIGO staging system classifies lymph node metastasis as IIIC stage, which indicates that lymph node metastasis can lead to a worse prognosis. Pelvic lymph node metastasis was classified as stage IIIC1 in general, according to the 2018 FIGO staging system; however, there was no more detailed staging. This study found that a positive common iliac lymph node was an independent risk factor for poor prognosis in recurrent cervical cancer patients, while other sites of pelvic lymph node metastasis are not. Therefore, further study is necessary to clarify the difference between the prognosis of patients with positive common iliac lymph nodes and those with positive internal iliac, external iliac, and obturator lymph nodes.

In addition, for cervical cancer patients with isolated lymph node recurrence, due to the difficulty of complete lymph node resection and the high risk of major vessel injury, there are few clinical data of salvage lymph node dissection. Salvage radiotherapy and/or chemotherapy are the main treatment after isolated lymph node relapse. Legge et al. (19) proposed that surgery is one of the most favorable options for gynecological cancer patients with simple lymph node recurrence, in which patients with ovarian cancer are the main beneficiary group. It has been reported that surgical resection of metastatic lesions limited to a single anatomical area outside the radiotherapy field, mainly involving para-aortic lymph nodes, is beneficial to the survival of patients (10). The advantage of surgical treatment is that the lesions that tolerate radiotherapy and chemotherapy or are not sensitive to radiotherapy and chemotherapy can be removed by surgery, so as to effectively improve the survival benefit.

This study indicated that the independent risk factors for poor survival after disease relapse in cervical cancer patients also included DM (DM alone/combined recurrence) and multiple site involvement, which is consistent with previous studies. Qiu et al. (13) evaluated the prognosis of 121 patients with recurrent cervical cancer after radical surgery and indicated that DM was significantly associated with a poor prognosis. Moreover, this study found that the incidence of LVSI was higher in patients with recurrent cervical cancer. This may be related to the fact that LVSI is more likely to lead to hematogenous metastasis at distant sites (20). Systemic chemotherapy as consolidation therapy in patients with LVSI may therefore reduce the incidence of DM.

This study is limited in that it was a single-institution retrospective study, which may have resulted in selection and time-trend bias, and the sample size was also relatively small.

## Conclusion

This study indicated that a positive resection margin, positive common iliac lymph node, para-aortic lymph node, no treatment after disease recurrence, early disease relapse, and DM were significantly associated with a poor prognosis in patients with recurrent cervical cancer.

Patients with high-risk factors and LVSI could receive targeted therapy, consolidation chemotherapy, or oral drug maintenance therapy after adjuvant CRT to reduce disease relapse and prolong survival time. The reexamination of patients could be started earlier, within half a year of the end of the initial treatment, which may help patients with early disease recurrence receive treatment earlier, potentially improving their prognosis.

## Data Availability Statement

The original contributions presented in the study are included in the article/supplementary material. Further inquiries can be directed to the corresponding author.

## Ethics Statement

The studies involving human participants were reviewed and approved by Liaoning Cancer Hospital & Institute ethics committee. The patients/participants provided their written informed consent to participate in this study. Written informed consent was obtained from the individual(s) for the publication of any potentially identifiable images or data included in this article.

## Author Contributions

H-TZ: Conception and design of the research, Acquisition of data, Writing of the manuscript. W-JY: Analysis and interpretation of the data, Statistical analysis. Y-HG: Critical revision of the manuscript for intellectual content. All authors contributed to the article and approved the submitted version.

## Conflict of Interest

The authors declare that the research was conducted in the absence of any commercial or financial relationships that could be construed as a potential conflict of interest.

## Publisher’s Note

All claims expressed in this article are solely those of the authors and do not necessarily represent those of their affiliated organizations, or those of the publisher, the editors and the reviewers. Any product that may be evaluated in this article, or claim that may be made by its manufacturer, is not guaranteed or endorsed by the publisher.

## References

[1] Jemal A Bray F Center MM Ferlay J Ward E Forman D. Global Cancer Statistics. CA Cancer J Clin (2011) 61(2):69–90. doi: 10.3322/caac.20107 21296855

[2] Bray F Ferlay J Soerjomataram I Siegel RL Torre LA Jemal A. Global Cancer Statistics 2018: GLOBOCAN Estimates of Incidence and Mortality Worldwide for 36 Cancers in 185 Countries. CA Cancer J Clin (2018) 68(6):394–424. doi: 10.3322/caac.21492 30207593

[3] Waggoner SE. Cervical Cancer. Lancet (2003) 361(9376):2217–25. doi: 10.1016/S0140-6736(03)13778-6 12842378

[4] Yang K Park W Huh SJ Bae DS Kim BG Lee JW. Clinical Outcomes in Patients Treated With Radiotherapy After Surgery for Cervical Cancer. Radiat Oncol J (2017) 35(1):39–47. doi: 10.3857/roj.2016.01893 27927011 PMC5398353

[5] Delgado G Bundy B Zaino R Sevin BU Creasman WT Major F. Prospective Surgical-Pathological Study of Disease-Free Interval in Patients With Stage IB Squamous Cell Carcinoma of the Cervix: A Gynecologic Oncology Group Study. Gynecol Oncol (1990) 38(3):352–7. doi: 10.1016/0090-8258(90)90072-S 2227547

[6] Sedlis A Bundy BN Rotman MZ Lentz SS Muderspach LI Zaino RJ. A Randomized Trial of Pelvic Radiation Therapy Versus No Further Therapy in Selected Patients With Stage IB Carcinoma of the Cervix After Radical Hysterectomy and Pelvic Lymphadenectomy: A Gynecologic Oncology Group Study. Gynecol Oncol (1999) 73(2):177–83. doi: 10.1006/gyno.1999.5387 10329031

[7] Peters WA 3rd Liu PY Barrett RJ 2nd Stock RJ Monk BJ Berek JS. Concurrent Chemotherapy and Pelvic Radiation Therapy Compared With Pelvic Radiation Therapy Alone as Adjuvant Therapy After Radical Surgery in High-Risk Early-Stage Cancer of the Cervix. J Clin Oncol (2000) 18(8):1606–13. doi: 10.1200/JCO.2000.18.8.1606 10764420

[8] Kim D Ki Y Kim W Park D Lee J Lee J. Adjuvant External Beam Radiation and Brachytherapy for Vaginal Resection Margin Positive Cervical Cancer. Radiat Oncol J (2018) 36(2):147–52. doi: 10.3857/roj.2018.00087 PMC6074069 29983035

[9] Benedet JL Odicino F Maisonneuve P Beller U Creasman WT Heintz AP. Carcinoma of the Cervix Uteri. Int J Gynaecol Obstet (2003) (Suppl 1):41–78. doi: 10.1016/S0020-7292(03)90115-9 14763169

[10] Legge F Chiantera V Macchia G Fagotti A Fanfani F Ercoli A. Clinical Outcome of Recurrent Locally Advanced Cervical Cancer (LACC) Submitted to Primary Multimodality Therapies. Gynecol Oncol (2015) 138(1):83–8. doi: 10.1016/j.ygyno.2015.04.035 25940427

[11] Gallotta V Conte C Federico A Vizzielli G Gueli Alletti S Tortorella L. Robotic Versus Laparoscopic Radical Hysterectomy in Early Cervical Cancer: A Case Matched Control Study. Eur J Surg Oncol (2018) 44(6):754–9. doi: 10.1016/j.ejso.2018.01.092 29422253

[12] Wang CJ Lai CH Huang HJ Hong JH Chou HH Huang KG. Recurrent Cervical Carcinoma After Primary Radical Surgery. Am J Obstet Gynecol (1999) 181(3):518–24. doi: 10.1016/S0002-9378(99)70486-2 10486457

[13] Qiu JT Abdullah NA Chou HH Lin CT Jung SM Wang CC. Outcomes and Prognosis of Patients With Recurrent Cervical Cancer After Radical Hysterectomy. Gynecol Oncol (2012) 127(3):472–7. doi: 10.1016/j.ygyno.2012.08.008 22902919

[14] Samlal RA van der Velden J Van Eerden T Schilthuis MS Gonzalez Gonzalez D Lammes FB. Recurrent Cervical Carcinoma After Radical Hysterectomy: An Analysis of Clinical Aspects and Prognosis. Int J Gynecol Cancer (1998) 8(1):78–84. doi: 10.1046/j.1525-1438.1998.09759.x 11576287

[15] Ansink A de Barros Lopes A Naik R Monaghan JM. Recurrent Stage IB Cervical Carcinoma: Evaluation of the Effectiveness of Routine Follow Up Surveillance. Br J Obstet Gynaecol (1996) 103(11):1156–8. doi: 10.1111/j.1471-0528.1996.tb09600.x 8917006

[16] Zhang MQ Liu SP Wang XE. Concurrent Chemoradiotherapy With Paclitaxel and Nedaplatin Followed by Consolidation Chemotherapy in Locally Advanced Squamous Cell Carcinoma of the Uterine Cervix: Preliminary Results of a Phase II Study. Int J Radiat Oncol Biol Phys (2010) 78(3):821–7. doi: 10.1016/j.ijrobp.2009.08.069 20207507

[17] Ng HT Kan YY Chao KC Yuan CC Shyu SK. The Outcome of the Patients With Recurrent Cervical Carcinoma in Terms of Lymph Node Metastasis and Treatment. Gynecol Oncol (1987) 26(3):355–63. doi: 10.1016/0090-8258(87)90027-8 3557197

[18] Joo JH Kim YS Nam JH. Prognostic Significance of Lymph Node Ratio in Node-Positive Cervical Cancer Patients. Med (Baltimore) (2018) 97(30):e11711. doi: 10.1097/MD.0000000000011711 PMC6078754 30045335

[19] Hong JH Choi JS Lee JH Bae JW Eom JM Kim JT. Laparoscopic Lymphadenectomy for Isolated Lymph Node Recurrence in Gynecologic Malignancies. J Minim Invasive Gynecol (2012) 19(2):188–95. doi: 10.1016/j.jmig.2011.10.013 22176995

[20] Viswanathan AN Deavers MT Jhingran A Ramirez PT Levenback C Eifel PJ. Small Cell Neuroendocrine Carcinoma of the Cervix: Outcome and Patterns of Recurrence. Gynecol Oncol (2004) 93(1):27–33. doi: 10.1016/j.ygyno.2003.12.027 15047210

